# Quantifying terrestrial carbon in the context of climate change: a review of common and novel technologies and methods

**DOI:** 10.1186/s13021-025-00316-1

**Published:** 2025-08-07

**Authors:** Samuel Gameiro, Manuel Eduardo Ferreira, Luis Fernando Chimelo Ruiz, Gillian L. Galford, Mojtaba Zeraatpisheh, Victor Fernandez Nascimento, Rosane Garcia Collevatti

**Affiliations:** 1 Remote Sensing and GIS Lab (LAPIG), Environmental Science Graduate Program, Federal University of Goiás, Goiânia, Brazil; 2 Rubenstein School of Environment and Natural Resources, University of Vermont, Burlington, VT 05405 USA; 3 Gund Institute for Environment, University of Vermont, Burlington, VT 05405 USA; 4 Research and Development (R&D) at Santos Lab Digital, Rio de Janeiro, Brazil; 5 Engineering, Modelling, and Applied Social Sciences Center, Federal University of ABC, Santo André, Brazil; 6 Genetics & Biodiversity Laboratory, Institute of Biological Sciences, Federal University of Goiás, Goiânia, GO Brazil

**Keywords:** Carbon stock, Remote sensing, Analytical techniques, PRISMA

## Abstract

**Background:**

Understanding carbon dynamics in Earth’s ecosystem is necessary for mitigating climate change. With recent advancements in technologies, it is important to understand both how carbon quantification in soil and vegetation is measured and how it can be improved. Therefore, this study conducted a bibliometric and bibliographic review of the most common carbon quantification methodologies.

**Results:**

Among the most widely used techniques, the Walkley-Black method and Elemental Analysis stand out for measuring below-ground carbon, while forest inventories are prominent for assessing above-ground carbon. Additionally, we found that the United States and China have the largest number of publications on this topic, with forest and agricultural areas being the most studied, followed by grasslands and mangroves. However, it should be noted that despite being indirect techniques, remote sensing, regression analysis, and machine learning have increasingly been used to generate geo-environmental carbon models for various areas. Landsat satellite images are the most widely used in remote sensing, followed by LiDAR digital models.

**Conclusions:**

These results demonstrate that while new technologies do yet not replace analytical techniques, they are valuable allies working in conjunction with the current carbon quantification process.

## Introduction

The global increase in carbon dioxide (CO_2_) and other greenhouse gas emissions drives changes to the climate, including warming temperatures and altered precipitation patterns [1]. Earth’s surface temperature, which has risen by almost 2 °C since pre-industrial times [2], could be stabilized by reducing CO_2_ concentrations in the atmosphere. Climate change mitigation, which involves reducing greenhouse gas emissions and enhancing the capture and storage (sequestration) of atmospheric CO_2_, requires deep understanding of carbon (C) dynamics, particularly within terrestrial ecosystems [3].

While the majority of C is stored in the oceans [4], human efforts to enhance C sequestration primarily target soils and vegetation– the second and third largest carbon sinks, respectively [5]. Thus, to help mitigate climate change, it is important to preserve or enhance terrestrial C storage, preventing it from being released into the atmosphere [6]. However, despite the global urgency for C sequestration, methods for its quantification remains inconsistent and uncertain.

Terrestrial C sequestration focuses on enhancing the storage of C within a natural carbon stock (CS) or pools. A CS refers to a system that can both store and release C, including components such as living biomass (both above- and below-ground), dead organic matter, or soil [7, 8]. Above-ground biomass includes all vegetation (e.g., trees, vines, understory, and herbaceous plants) [9, 10], while below-ground biomass includes all living, dead roots, soil fauna, and soil microbial communities [10]. The carbon content in biomass encompasses the total C content found in above- and below-ground biomass of organic materials [7, 11]. Soil carbon (SC) can be either inorganic or predominantly organic, while soil organic carbon (SOC) specifically originates from the decomposition of animals, plants, or microorganisms, as well as from carbonized and humidified materials [12]. Carbon sequestration refers to the annual rate of change in C storage; a positive rate indicates the presence of a C sink [13, 6].

Quantifying CS is essential for developing climate change adaptation and mitigation strategies, including creating models for simulation scenarios to support management and decision-making [10]. Vegetation CS is integrally linked to biomass, especially above-ground, and is directly affected by degradation and deforestation [3]. Vegetation CS is generally quantified using forest inventory methodologies and allometric equations [14, 7, 10, 15, 16] However, for accuracy it is important to quantify the C in the soil itself, especially in its surface portion, which has greater concentrations of biomass and C [7, 17].

The main methodologies for quantifying SOC are elemental analysis (EA), Walkley-Black (WB), or variations of these [18, 19]. Despite this, new methodologies have been developed and applied over the last 15 years, such as spectrometric analysis [20, 21], remote sensing [22, 23], geo-environmental modeling [24, 25], statistical modeling using regression [26, 27], and machine learning. When integrated with field measurements and environmental variables, these approaches have produced substantial advances in SOC estimation. For example [28, 29], used integrated methodologies and demonstrated the effectiveness of this combination for more comprehensive SOC estimates.

However, even with these technologies, quantifying SOC remains a challenge in most places due to the complexity and cost of many measurement approaches [23, 30]. While these newer techniques enable rapid and cost-effective SOC predictions, their repeatability and accessibility varies depending on regional and ecosystem-specific factors. For example, SOC estimation errors tend to be higher in tropical forests than in temperate regions [17]. An in-depth investigation of terrestrial SC and sequestration– particularly SOC– requires accurate and consistent long-term field measurements, synthesized with methodologies and data collection protocols [31, 17]. The development of rapid, economical, effective, and accurate techniques remains crucial [21, 1].

In this study, we carried out a bibliometric analysis of carbon quantification techniques implemented since 2000 and a bibliographic evaluation of the most widely-used methodologies for quantifying carbon in soil and vegetation. We highlighted their importance for addressing key gaps and future potential in the context of the current global climate change scenario. Additionally, we examined the land uses and land covers most frequently studied in relation to organic carbon.

## Methodology

### Bibliometric review

**Fig. 1:**
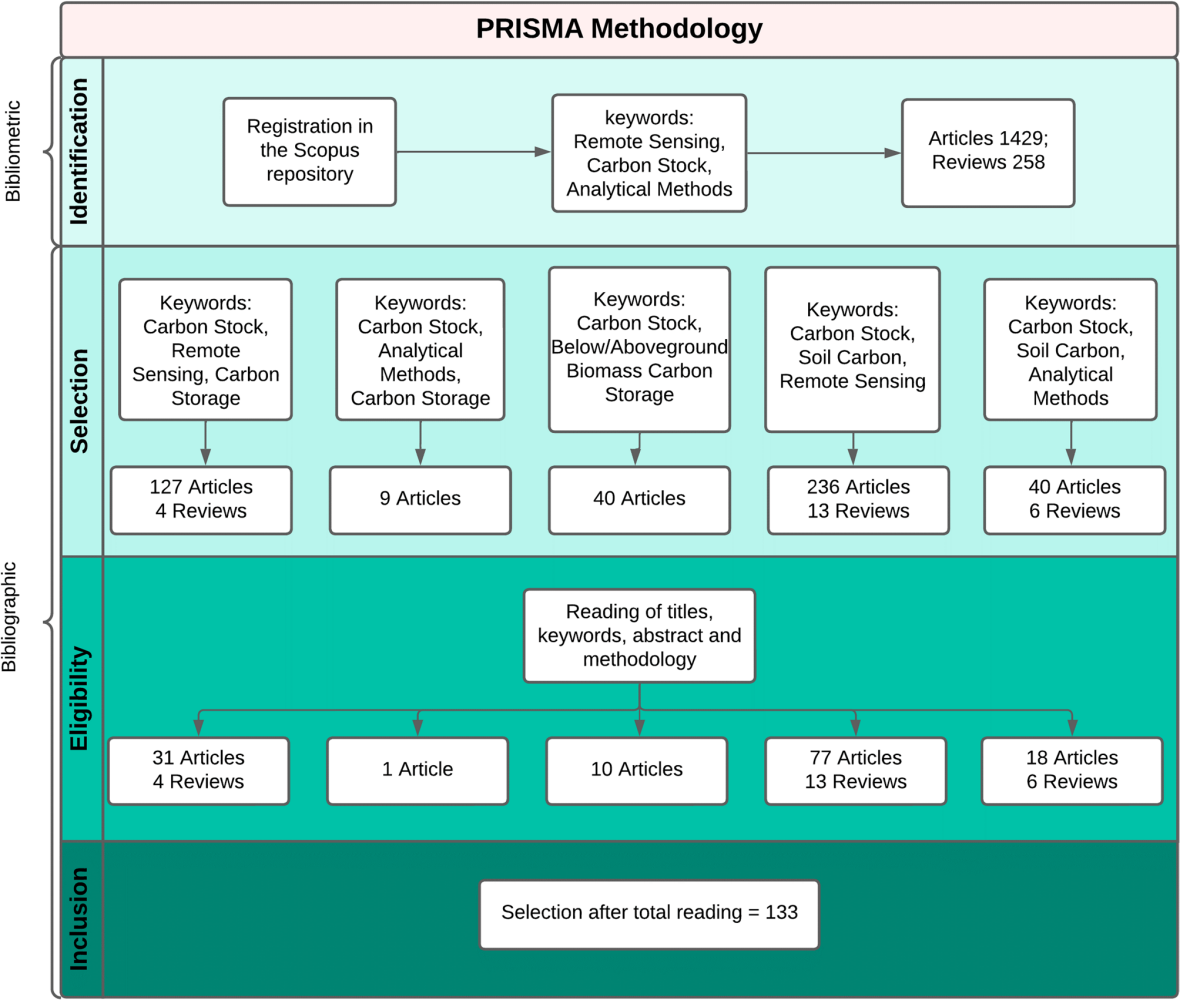
Flowchart of the prism methodology, showing the bibliometrics and bibliography phase, the words and techniques used and the number of articles found in each phase

We used the Scopus database for bibliometric review because it is the largest peer-review database and overlaps with other key resources, such as the Web of Science. Using the keywords “Carbon Stock,” “Remote Sensing,” and “Analytical Methods,” we limited results to articles and reviews published in English, between 2000 and 2023, and in open-access journals that have greater visibility and more accessibility. With this criteria, we found 1429 articles and 258 reviews (Fig. 1). To analyze and visualize bibliometric and scientometric relationships, we used VOSviewer [32], which provides robust tools for bibliometric network visualization through co-occurrence, cooperation, and co-citation analyses [33]. It also calculates the Total Link Strength, a key metric that quantifies the strength of connections between nodes, such as keywords, authors, or institutions, highlighting collaboration and thematic relationships in the research field [34, 35]. We assessed keyword frequency, number of articles published per journal, number of citations for each journal, and nationality and number of publications of each author. Cluster maps were generated for keywords used more than 50 times to analyze the strength of the relationships between keywords. We also clustered countries with more than 10 published works to understand the relationships between leading researchers in these countries.

### Bibliographic review

For the bibliographic review, we used the Preferred Reporting Items for Systematic Reviews and Meta-Analyses (PRISMA) methodology [36]. In the identification phase, the search results were used to estimate the number of articles on the subject and to determine which keywords could be used for a more detailed analysis. In the selection phase, we used several keywords to select better articles focused on C quantification and analytical and remote sensing techniques. In the eligibility phase, we read and selected the articles that most closely matched and conveyed relevant information on C quantification methodologies. Finally, 133 articles were included for bibliographic review. We extracted terminology, methodology, and techniques used for C quantification from these articles.

Based on the selected articles, we conducted an analysis to determine which land use land cover (LULC) classes were studied most frequently, and specified whether they were primarily associated with soil attributes or vegetation types. Additionally, we systematically identified and categorized the key remote sensing terminologies (e.g. satellites and sensors), data sources (e.g., platforms, spectral libraries), modeling techniques (e.g. regressions, machine learning, or geo-environmental models), and analytical methods (e.g., laboratory techniques) that were applied across all studies.

## Results

### Bibliometric statistics

**Fig. 2:**
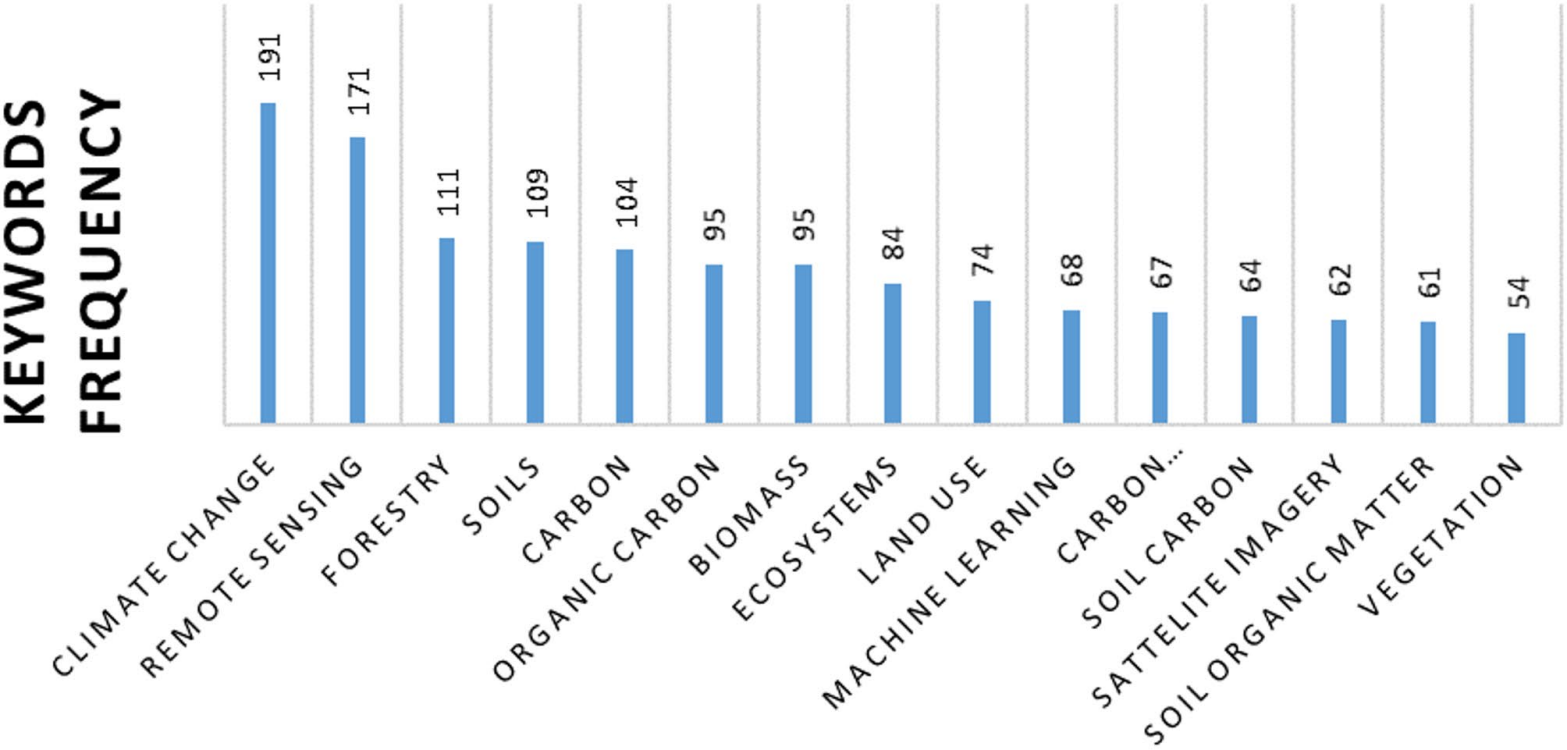
Fifteen of the most frequent keywords and the number of mentions found in the 1429 articles and 258 reviews

Through the bibliometric review, we identified 1429 articles and 258 reviews that used the keywords “Carbon Stock,” “Remote Sensing,” and “Analytical Methods” published in English in open-access journals between 2000 and 2023. Among these, 15 keywords appeared more than 50 times (Fig. 2). “Climate change” was the most frequently mentioned term, appearing 191 times, followed closely by “remote sensing” with 171 mentions, and “forestry” with 111 mentions.

**Fig. 3:**
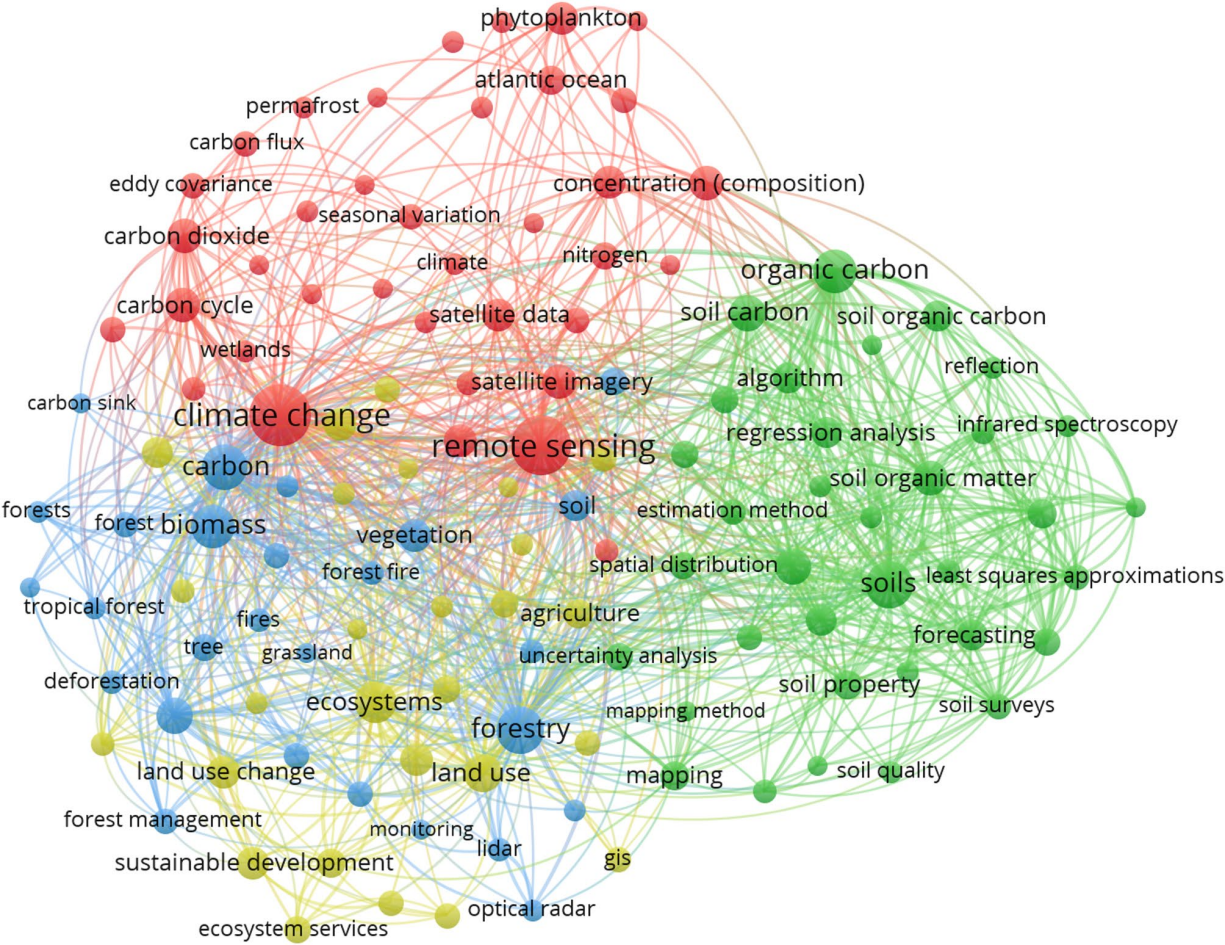
Keywords occurring more than 20 times in the bibliometric analysis. The color-coded clusters represent groups of co-occurring terms, highlighting thematic connections and research trends across the analyzed articles

Certain keywords are related to each other and frequently appear together in scientific literature (Fig. 3). The word “climate change,” often associated with “remote sensing,” “carbon cycle,” “satellite imagery,” “satellite data,” and “wetlands,” points to the increasing use of space-based technologies to monitor the impacts of climate change on ecosystems and the global carbon cycle. This suggests that an important focus of research is on remote monitoring to assess how different environments (especially wetlands) contribute to or mitigate carbon emissions.

The term “remote sensing” is also related to “estimation method,” “spatial distribution,” “soil carbon,” and “vegetation,” indicating that research often uses remote sensing to map the spatial distribution of carbon in both soils and vegetation. This approach highlights the importance of advanced technologies to study carbon on a large scale and assess its dynamics in different ecosystems.

The connection of forestry with words such as “biomass,” “carbon,” “LiDAR,” “optical radar,” and “land use,” reflects the use of modern technologies, such as LiDAR, to monitor forest biomass and stored carbon. This also demonstrates a concern about the impact of land use on carbon dynamics and the role of forests as carbon sinks.

The association of soils with “soil properties,” “soil organic matter,” “organic carbon,” “infrared spectroscopy,” “soil carbon,” and “mapping” highlights a strong interest in the detailed characterization of soils and their capacity to store carbon. The use of spectroscopy and mapping suggests the search for precise methodologies to quantify and monitor organic carbon in the soil.

**Fig. 4:**
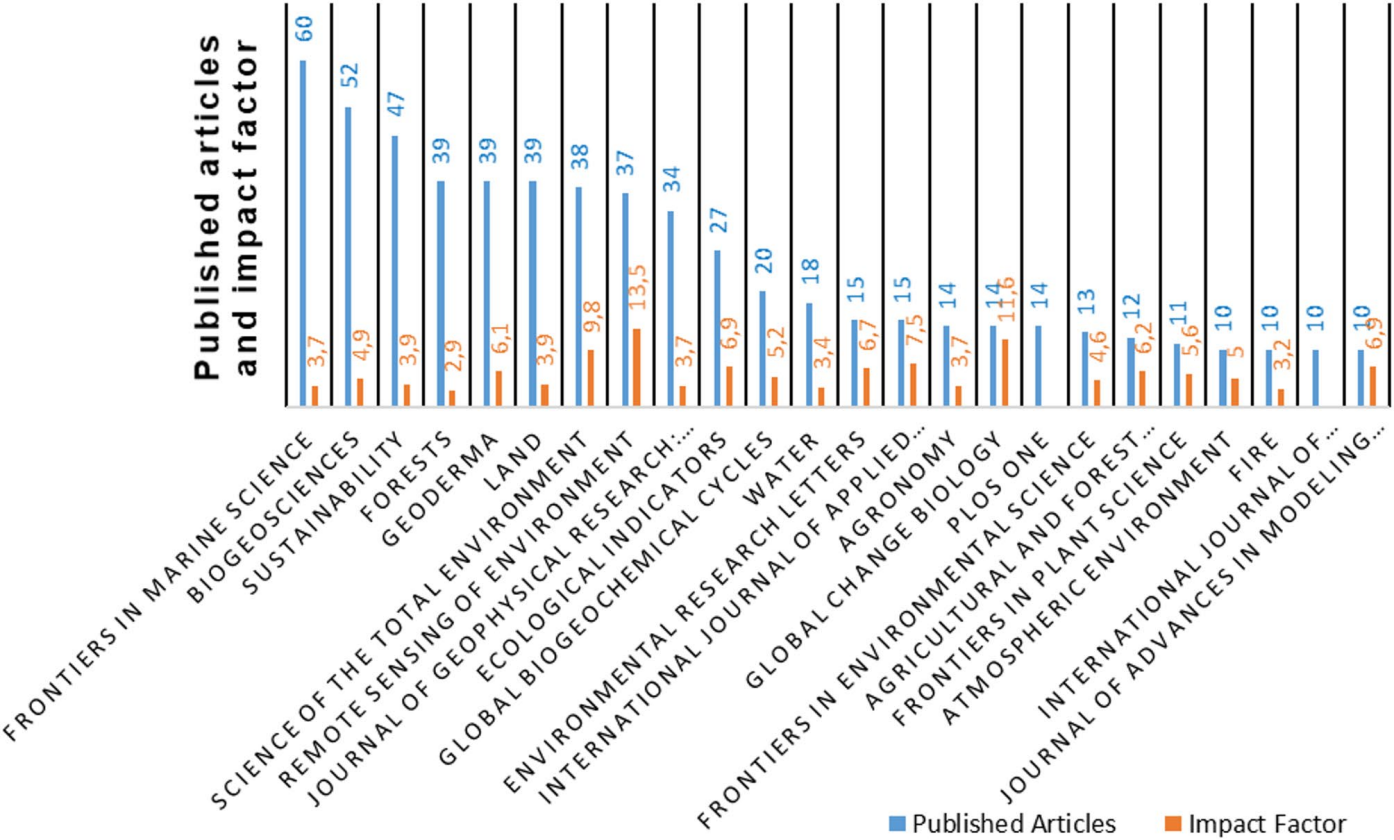
Number of articles published by journals with ten or more publications in the bibliometric review, accompanied by their respective impact factors, illustrating publication volume and journal influence in the field

*Frontiers in Marine Science* was the journal with the highest number of publications (60 articles) and impact factor (IF) 3.7 (Fig. 4). It was followed by *Biogeosciences*, with 52 articles and IF 4.9, and *Sustainability*, with 47 published articles and IF 3.9.

**Fig. 5:**
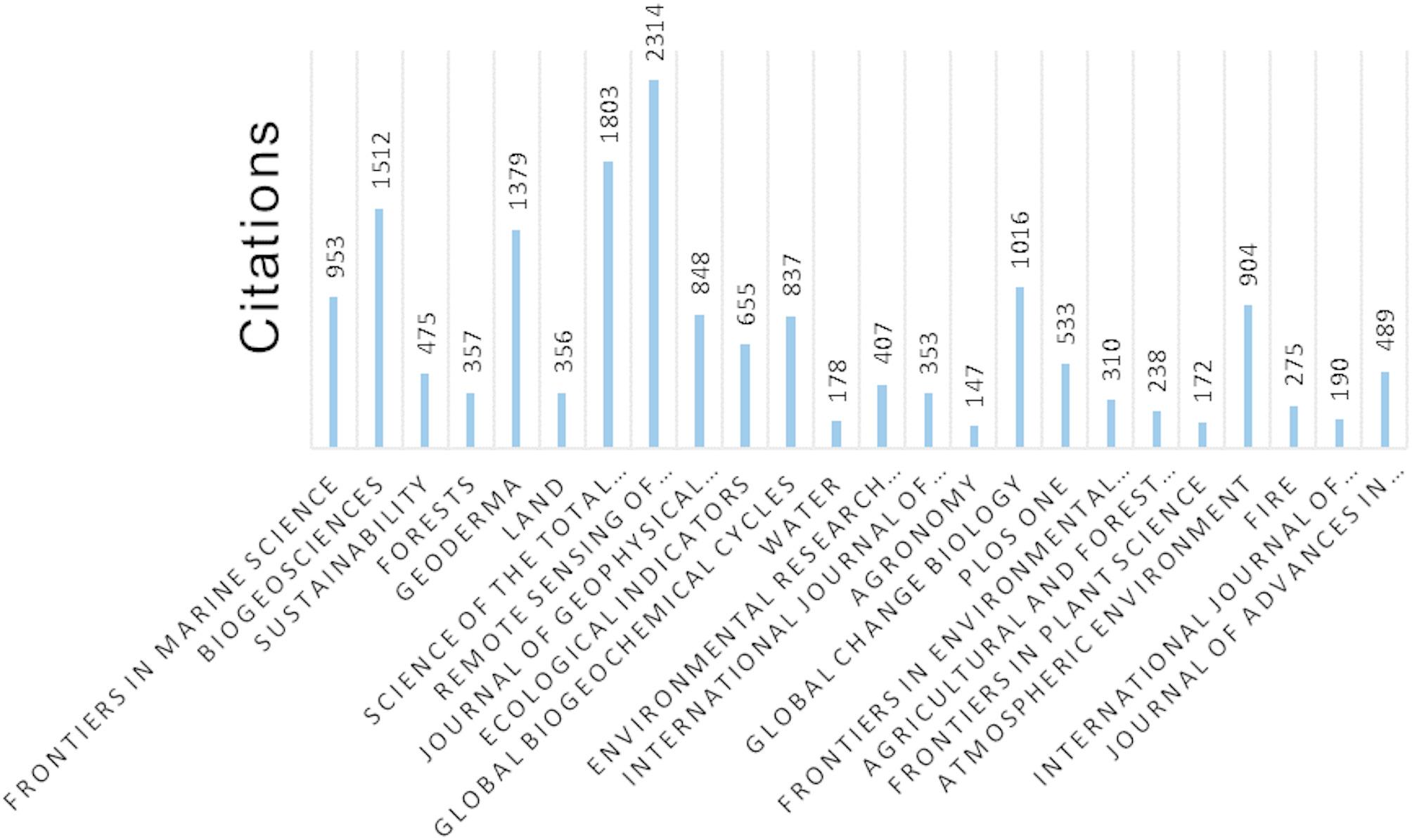
Total citations of articles published by journals with more than 10 publications in the bibliometric review, showcasing their academic influence and contribution to the field

Among the top 20 journals with the most publications identified in the bibliometric review (Fig. 5), *Remote Sensing of Environment* had the highest number of citations (2,314), followed by *Science of the Total Environment* (1,803) and *Biogeosciences* (1,512).

**Fig. 6:**
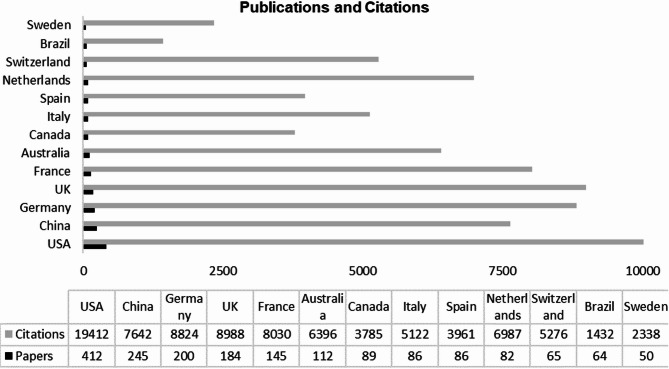
Countries with more than 50 published articles and their corresponding citation counts, highlighting the academic impact and research contributions of each nation within the bibliometric review

Scientists from the United States of America (USA) published the largest number of articles (412, Fig. 6) and garnered the most citations (19,412). Researchers from China published 245 articles, and those from Germany published 200. However, Germany had 8,824 citations, while China received 7,642. As expected, given its high publication volume, the USA also had the most international collaborations, particularly with Canada, the United Kingdom (UK), Germany, Brazil, and France. In contrast, China’s research network was more connected among other Asian and Middle Eastern countries, with strong connections to India, Pakistan, Iran, and Japan.

### Direct quantification techniques

**Fig. 7:**
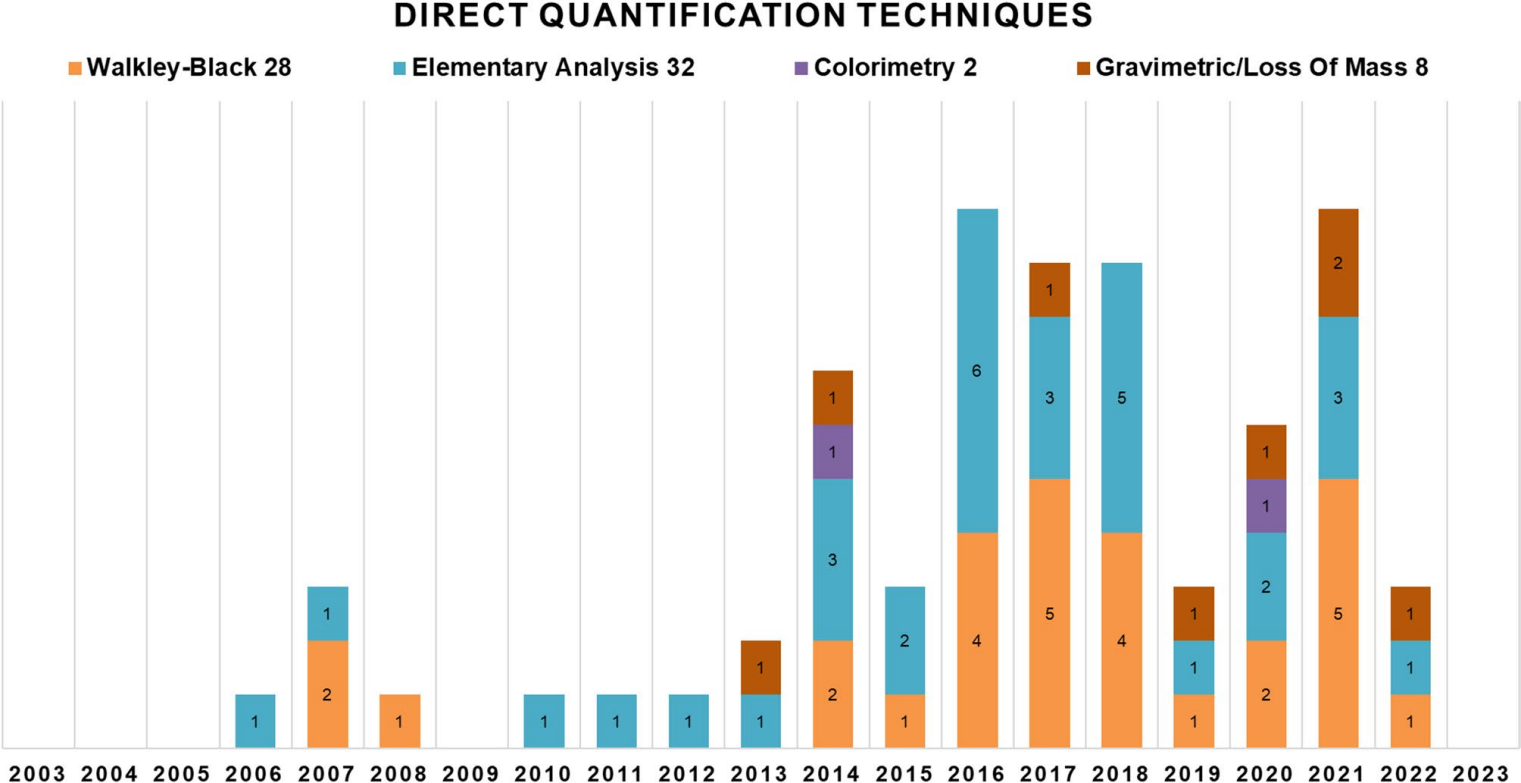
Primary direct methodologies for quantifying above- and below-ground carbon from 2003 to 2023, along with the number of articles employing each technique

The most common direct C quantification techniques found in the literature (Fig. 7) include the WB method [37], colorimetry [38], EA, and mass loss for below-ground carbon. For above-ground carbon, the most widely used technique is the mass loss technique.

For below-ground carbon, the WB and EA methods are used most frequently and yield similar results, making them widely regarded as standard techniques for this type of analysis. For above-ground carbon, the mass loss technique can be applied, however it is less commonly used due to its destructive nature and labor-intensive process.

#### The Walkley-Black method and colorimetry

The WB method is still one of the most widely used techniques for quantifying C used in soil laboratories, despite the environmental problems generated by its chemical reagents [18]. The method consists of oxidizing soil’s organic C using a potassium dichromate and sulfuric acid mixture. The original methodology, without external heating, cannot measure 100% of the organic C in the sample. Thus, different conversion factors estimate the amount of organic C in the samples [39]. These conversion factors are based on the type of soil and organic matter estimated amount, and sampling site geomorphology. Because of these environmental and mathematical disadvantages, several modifications have been made to the methodology, such as adding a small amount of silver sulfate to the solution [40, 41] or using the Mebius methodology [42], which follows the same procedure, but an external flame is added for a certain period to cause the organic C to burn completely and eliminate the need of correction factors.

The colorimetric method is similar to WB and is also used in laboratories. It differs from WB by using sodium dichromate instead of potassium, and the result is no longer obtained by titration but by spectrophotometry. The colometric method also needs no external heat source [43], making it a feasible alternative for soil analysis with a high correlation (86%) with the EA method as a reference [44].

#### Elementary analysis - CHNS/O

The dry combustion EA method is widely to be the standard methodology for quantifying C in soil [19]. It consists of burning the samples at high temperatures and measuring the percentage levels of each element present in the samples using a thermo-conductivity sensor [19]. The most significant advantages of this methodology are the speed, precision, and reliability of the analysis. However, the cost of acquiring an EA device is very high, making the process unfeasible in many cases [18, 17]. state that, despite the high cost of the apparatus, this is the most suitable method for carrying out C analysis because it has the highest accuracy, decomposes all forms of C, requires a small sample, and is a high-speed method. Because it is considered to be standard methodology and is, to date, the most accurate of all, EA is often used as a reference and calibration method, as can be seen in [45, 46].

#### The gravimetric or loss of mass by ignition method

The loss of mass by ignition methodology estimates C content with a dry sample. The C content is calculated based on the initial weight measured compared to the final weight of the sample after the incineration process [38]. The mass lost at the end of the sample is attributed to organic matter, from which C is derived, of which approximately 58% of the organic matter as C can be attributed [47]. Although it has the advantage of not using any chemical reagent, time spent on analysis and the wide range of temperatures for ignition are important obstacles to applying this technique [48, 49]. Soil mineralogy can significantly affect the results obtained by this method, especially soils with large amounts of gibbsite, which has a positive relationship with C [19]. Soil type, depth, temperature, and the nature of the soil’s organic C can also affect the results [17]. Moreover, C content can be wrongly estimated in soils with large amounts of organic matter [50].

### Indirect quantification techniques

**Fig. 8:**
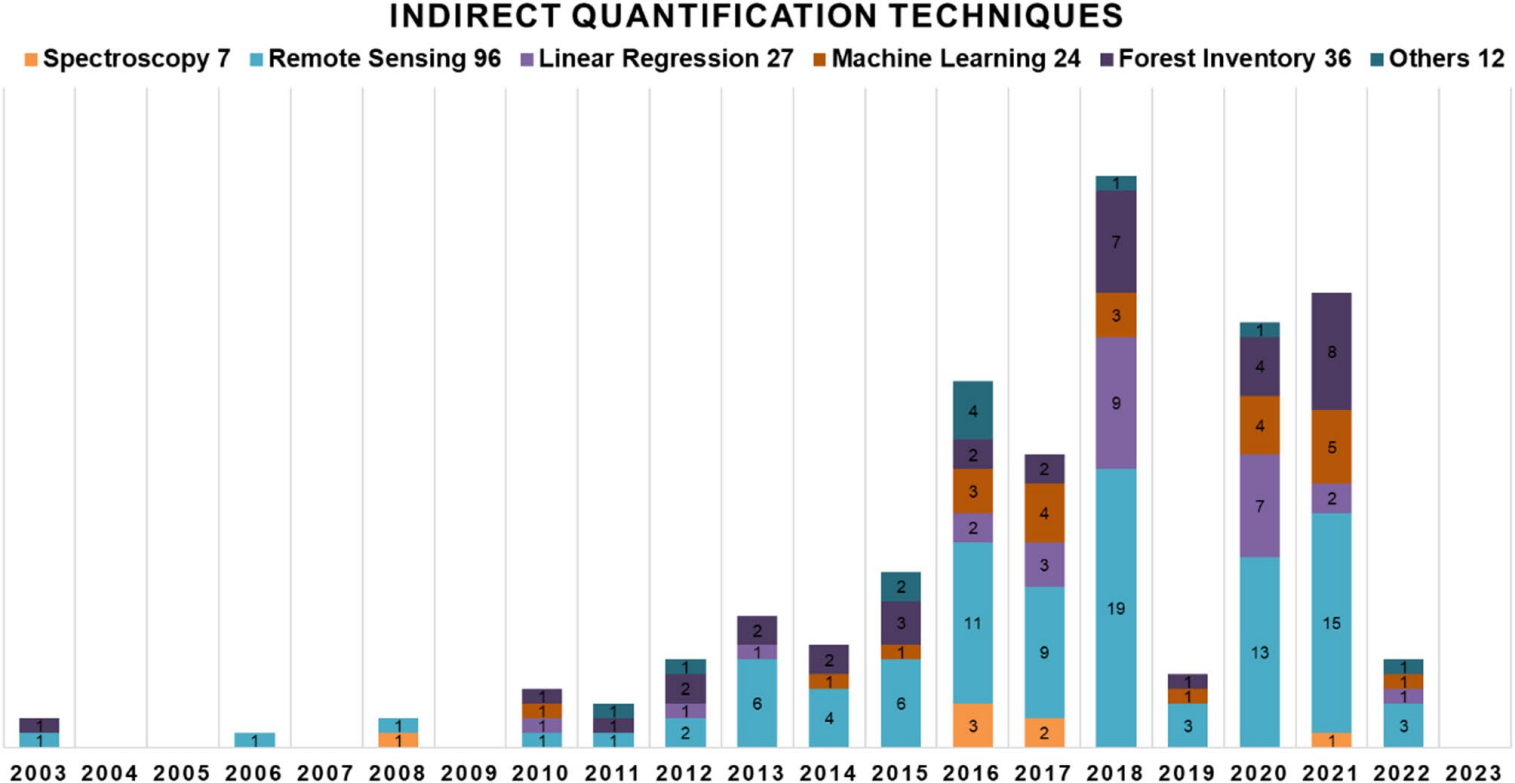
Indirect methodologies for quantifying above- and below-ground carbon from 2003 to 2023, with the number of articles employing each technique

In indirect methodologies, the main techniques used were remote sensing, linear regression, machine learning, spectroscopy, and environmental models such as InVEST, RaCSA, Century and Yasso07 (Fig. 8). Remote sensing has regulaly been used in the last 20 years, with publications becoming more frequent in 2013, and reaching their peak in 2018. Along with remote sensing, use of regression and machine learning techniques have also increased in use since 2016. Geo-environmental models featured prominently in 2016, but hardly at all in the years following.

#### Spectroscopy

The spectroscopy method is a fast and cost-effective technique for identifying and quantifying organic C [17], which can be used directly in the field, in the laboratory, and in manned or unmanned aircraft [51]. The visible spectrum (Vis, 400–700 nm), near-infrared (NIR, 700–900 nm), and shortwave infrared (SWIR, 900–2000 nm) spectroscopy methods are the most widely used because of their high sensitivity for detecting organic and inorganic soil components [52]. However, devices with a mid-infrared spectrum (MIR, 2500–25000 nm) are also used because they can obtain more information than presented in the Vis-NIR-SWIR spectrums [21].

#### Remote sensing

Remote sensing to quantify C is an indirect methodology and usually requires additional data, such as vegetation structures [53]. It has become an essential tool for C estimation because it is free, and high-quality data can be updated continuously [54]. It is used with different platforms, such as satellites, aircraft, UAV or drones, transporting different types of sensors, which can be optical, laser, or radar. The biomass and C detected by remote sensing can also be extrapolated to larger areas using linear regressions, clustering techniques, and machine learning methodologies [5].

#### Regression and machine learning techniques

Regression models calculate the relationship between predictive variables, such as precipitation, land use, temperature, and extensive data extracted by remote sensors with biomass or C data [5, 22]. In addition to regression models, machine learning algorithms offer very efficient statistical modeling for C estimation due to information learning between predictor and response variables [55].

#### Forest inventory/allometric equations

Forest inventory involves collecting specific information about the woody plants in a given area. It is possible to quantify biomass and C using allometric equations, which portray an empirical relationship between biomass or C content and plant allometry information measured in field inventories [56]. These equations use vegetation height and diameter at breast height as the main variables, which are the simplest to measure in the field. They also use variables obtained in the laboratory, such as volume, leaf area [5], and basal area, which, according to [16], is a crucial element in C quantification since it is directly linked to height.

#### Other techniques

The “other techniques” category includes models that address different environmental and economic aspects related to carbon. One example is CO2FIX, which is a dynamic model designed to simulate the carbon cycle in forests, plantations, and agroforestry systems [57]. It calculates carbon capture in different components of the ecosystem, such as above-ground biomass, roots and soil, as well as considering wood products. The model also makes it possible to analyze forest management scenarios and their implications for carbon sequestration, and is widely used to evaluate climate change mitigation strategies. The Century model simulates nutrient flows in the soil and the long-term storage of organic matter. It is especially useful for estimating soil carbon stocks [58], considering processes such as decomposition, and interaction with vegetation. And the InVEST model (Integrated Valuation of Ecosystem Services and Tradeoffs) is designed to evaluate ecosystem services, such as carbon storage and sequestration. It combines environmental and economic factors, making it possible to estimate the value of these services under different land use scenarios [59]. These models are essential tools for estimating terrestrial carbon, each with specific approaches and distinct focuses. They provide critical support for decisions related to climate change mitigation and the sustainable management of ecosystems.

### Land cover classes

**Fig. 9:**
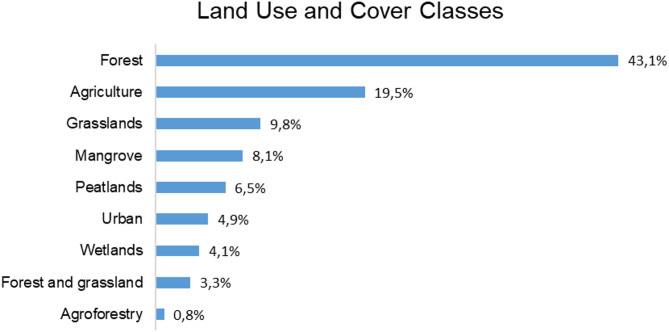
Land use and cover classes found in the bibliographic review of 133 articles and their respective mentioned percentage (total = 100%)

The articles included eight different classes of land cover: forests, agriculture, grasslands, mangroves, peatlands, urban, wetlands, and agroforestry (Fig. 9). In addition, only 3.3% of studies compared different land cover classes, particularly forest and grassland.

Forest is the most frequent land cover (43.1%) studied in the articles, primarily tropical forests [60, 61, 15], bamboo forests [25, 22], boreal forests [62, 63], and planted forests [64]. Agriculture is the second most studied (19.5%). Each of the other land cover classes accounted for less than 10% of the articles analyzed. For example, grasslands were studied in 9.8% of the articles, mangroves in 8.1%, and peatlands in 6.5%. Despite forests being the largest LULC in the articles researched, several articles deal with below-ground carbon in forest environments, agriculture, grassland, etc.

## Discussion

### Bibliometric analyses

Climate change has become a significant topic in both academic and non-academic communities over the last few decades, and has been featured prominently in newspapers, reports, research, and social media. This may explain why it is the most frequent keyword in articles and connected to keywords such as “C cycle,” “carbon dioxide,” and “C flux,” as C is critical to global warming, climate change mitigation and adaptation. Remote sensing is a promising methodology to estimate and predict C flows that is also cost-effective [20]. This explains why remote sensing and climate change are highly connected, in addition to keywords such as “satellite imagery” and “satellite data.” This analysis also suggests that remote sensing is enabling new, emerging fields of research such as digital soil mapping or climate modeling.

“Forestry” and “soils” were two other recurring keywords, indicating that these ecosystems are frequently studied for C quantification. This is expected since they are the major C-storing terrestrial ecosystems [5]. Besides a connection with keywords such as SC, organic C, and SOC, the term “soils” is also connected with specific and highly used methodologies for C quantification—such as infrared spectroscopy, algorithm, and regression analysis—suggesting that statistics and computer models are widely used in SOC research [61, 65, 66]. On the other hand, the keyword “forestry” is connected with two different C research techniques, LiDAR and radar [62, 66], due to their ability to penetrate and estimate the height and vertical structures of vegetation [29]. The different connections and relationships between words involving the same theme demonstrate how various methodologies are used to quantify C and show which are more interconnected with above-ground and below-ground C.

Although the journals *Frontiers in Marine Science*, *Biogeosciences*, and *Sustainability* published the most articles in this area, they were not the most cited, likely because they address more specific topics on CS, such as biogeochemistry, soil nutrition, and effects of soil chemical properties on CS. On the other hand, the most cited journals, *Remote Sensing of the Environment* and *Science of the Total Environment* include empirical studies from diverse countries and ecosystems, showing the application of C quantification methodologies in several types of soil and vegetation. It should also be noted that the latter two journals with the largest number of citations also had high impact factors: 13.5 for *Remote Sensing of the Environment* and 9.8 for *Science of the Total Environmental*.

The USA has the most publications and the largest network of relationships with other countries that publish in this area, including Brazil and South Africa, the United Kingdom, and France. This reinforces the USA’s critical role in developing academic research, technology, and information about climate change. It is important to note that these results may be biased because we limited our search to English-language journals, which tend to be dominated by native English researchers and exclude many regional journals in other languages.

China has the second-highest number of publications on the subject of C. Interestingly, together with the USA, China emits the largest quantities of greenhouse gases [67]. Although China has relationship networks with many countries, like the USA, it has more collaborations with Asian and Middle Eastern countries such as India, Iran, and Japan. It is also worth noting that of the 13 countries with the most publications, only Australia and Brazil belong to the Southern Hemisphere. This shows that despite extensive cooperation among researchers from different countries, investments in scientific research and work produced are concentrated mainly in the Northern Hemisphere.

Of the 133 articles analyzed, 107 detailed their study area and only 36.38% of these areas were in the southern region of the globe, with Brazil and Australia standing out. Despite the large amount of forest present in the Northern Hemisphere, especially in the more polar regions, the biggest biodiversity hotspots are in the Southern Hemisphere [68], making them priority areas for studies that can help conserve them. However, there has been limited financial investment in research infrastructure and studies conducted in the Southern Hemisphere.

### Quantification techniques

#### Above and below-ground carbon with direct methodologies

Laboratory techniques are used for calculating below-ground organic C. The most commonly used techniques are WB, which uses a wet combustion methodology, and EA, which uses a dry combustion methodology. We found no consensus in the literature on which technique is the standard for studying C. Some authors state that WB is the most widely used [19, 69], while others claim that the main technique used is EA [43, 23, 70].

The difference in cost is one of the primary deciding factors between these two methodologies that can influence scientists’ choices. WB methodology involves common reagents, and no specific equipment maintenance by specialists is necessary. On the other hand, the EA methodology requires sophisticated equipment and technology, as well as human resources for maintenance, cleaning, and training, which can increase the analysis costs. Equipment calibration for EA and handling samples, and reagent amounts used in WB are critical points in the methodologies that can result in significantly different costs across studies.

Sample handling while applying the techniques can also vary results. WB consists of a manual method, which can be influenced by the technician in charge of the operation, while EA has a more automated process. This operational factor can also result in differences between the two methodologies. Although [45] obtained similar organic carbon results when utilizing both methods [18], obtained 17.7% lower values in WB than EA [19]. also compared the two methodologies and found better results with the use of EA, highlighting that the process promotes the complete oxidation of carbon, whether organic or inorganic, and is considered a benchmark for total carbon analysis.

In addition to WB not being able to completely oxidize carbon, which can lead to inaccurate results, it has the problem of using toxic reagents (K₂Cr₂O₇ and H₂SO₄) in the procedure. Although the colorimetry technique presents the same problem of not fully oxidizing carbon, like most wet combustion methodologies, it offers an alternative to WB by replacing K2Cr2O7 with Na2Cr2O7, making the process less harmful to the environment while obtaining similar results [19, 43]. Therefore, although WB is still one of the most widely used methodologies for below-ground C quantification, new, similar, and cleaner methodologies can be used that have less impact on the environment.

The loss of mass through the ignition method is also used to calculate organic matter and the level of organic C using conversion factors, which consist of the amount of C generally present in organic matter. The conversion factor can vary according to the composition of the matter or the type of soil, as seen in [71, 47], who used a conversion factor of 0.58, versus [72] used a conversion factor of 0.47 in Bangladesh soils [47]. claim the loss of mass by ignition is the best way to calculate C in organic soils.

#### Above and below-ground carbon with indirect methodologies

##### Spectroscopy

Spectroscopy has also been used for below-ground C quantification, although more modestly. It is a fast, easy-to-use, and cost-effective technique for analyzing chemical elements in soil [21]. It requires minimal preparation, making the analysis process more accessible and agile [45]. Analysis using a spectroradiometer usually provides results similar to those obtained using standard laboratory methodologies [45], making it an essential tool for the future. The efficiency and speed of analysis are likely to increase significantly because of technological improvements such as handheld spectroradiometers, which can be used directly in the field, and the possibility of performing spectroscopy by aircraft [51]. However, for now these remain high-cost techniques [17]. Spectrum technology is expected to increase with the emergence of soil spectral libraries, where data can be used directly or to provide calibration for C estimation models. The Australian National Library already has more than 500 soil spectral samples [73], and the Brazilian Soil Spectral Library continues growing [74].

Because it is easier to obtain data for Vis-NIR-SWIR technology, its interpretation is already better known and more detailed as a methodology. Additionally, because of the cost-benefit comparison of the technique and its instrumentation, Vis-NIR-SWIR technology is the most suitable for measuring SOC concentration as compared to MIR and Laser Induced Breakdown Spectroscopy (LIBS) [52, 20, 46]. used the Vis-NIR-SWIR spectrum to analyze C in mangrove ecosystems and, despite obtaining excellent results, made it clear that using the range between 600 nm and 1000 nm is sufficient to get acceptable results. Despite this, it cannot be assumed that this section of the electromagnetic spectrum is adequate for analysis in other locations with different geo-environmental characteristics, where in some cases, it may be necessary to use MIR.

[21] used two different spectrometers, one in the Vis-NIR-SWIR range and the other in the MIR range, to predict C dates and organic matter in soils in Iran. Using the same spectrum bands [75], were able to predict C and nitrogen in soils in northeastern Brazil. According to [70], using the MIR spectrum band, coupled with modeling techniques, can be viable for accurately measuring organic C on a large scale and in a cost-effective manner [45]. compared the use of the MIR spectrum with the EA and WB methods. They obtained satisfactory results for both, with the advantage of not needing to make sample preparations and the possibility of using more variables present in the MIR spectrum. These studies show the remarkable capacity of spectroscopy techniques to obtain results equivalent to those obtained with analytical techniques such as WB and EA while being more cost-effective, primarily because they do not require complex pre-processing of samples.

[52] warn that one of the biggest problems with spectroscopy sensors is the need for multivariate calibration of the equipment and the lack of spectral data and libraries. They also state that despite all the advances in spectroscopy systems, there is a need to create sensors that are less dependent on calibration and that are safer, cheaper, and more accurate [46]. also confirm the dependence on devices and the lack of spectral data, especially for mangroves, pointing out that mangroves around the world can have different spectral characteristics, as can regions with different forest types.

Although it doesn’t stand out in the studies, LIBS uses atomic emission spectroscopy, which consists of a laser pulse that heats the soil sample. The emission spectrum of the sample is analyzed in a spectral range from 190 nm to 1000 nm [52]. The easy sample handling, analysis speed, and potential application of this technology in portable field devices are the main advantages of LIBS [76]. However, effectively and consistently using this technology continues to face challenges, including controlling the plasma generated by the laser, its interaction with the environment, and the influence of the physical and chemical structures of the samples [76]. In Amazonian soils, LIBS provided detailed information on soils chemical composition [77]. Overall, LIBS has smaller prediction errors compared to Vis-NIR spectroscopy, although both technologies have similar results [78].

##### Remote sensing

**Fig. 10:**
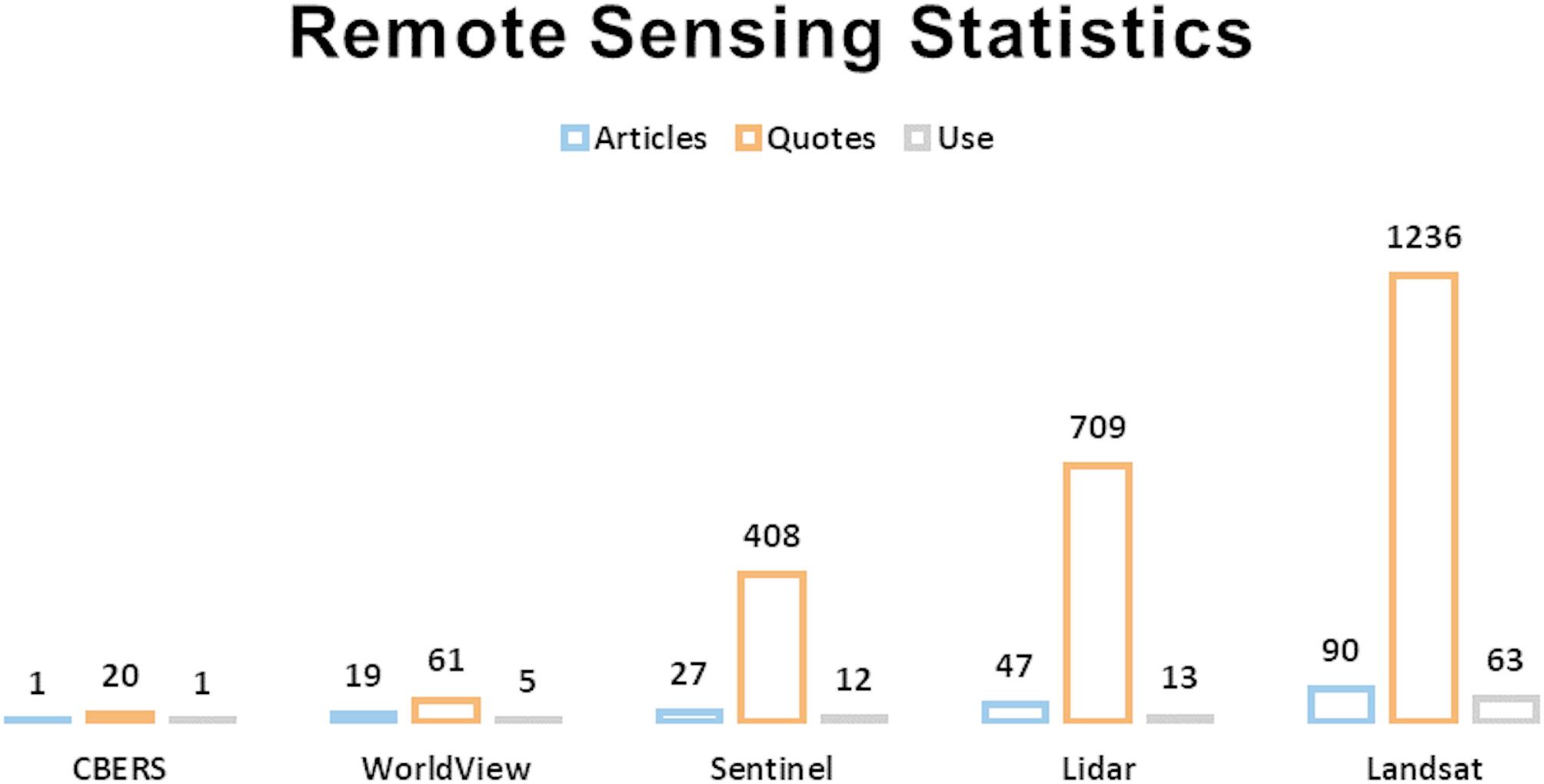
Statistics on the most utilized and referenced satellites and sensors, presented through metrics including the number of articles, citations, and usage

Remote sensing can quantify both above- and below-ground C, but its accuracy is limited, causing high rates of uncertainty [17]. Remote sensing uses satellite images as its primary tool in C quantification in most studies surveyed [65, 54, 27]. Satellite images are an essential tool for assessing and monitoring CS, especially in remote areas, and they also allow data to be extrapolated [79]. Figure 10 shows the satellites and sensors most commonly found in the articles reviewed. It breaks down the data into the number of articles about the sensor satellite, the number of times it is mentioned in the text (quotes), and how many articles used the sensor.

The Landsat series is the most often cited and most frequently used satellite data. This satellite series uses multispectral sensors, has an extensive collection of free images, and has excellent potential for historical studies due to its images dating back to the 1980s [27]. Despite this, the series also has some disadvantages, including images in medium resolution and obscured by clouds, making this satellite unable to work in certain areas of study or with greater data precision [80]. However, Landsat still has the longest collection of satellite images that can help us understand how historic C flows in different ecosystems.

Sentinel images are also widely used. They have two major advantages over Landsat images: better special resolution and radar images, which are not affected by clouds, in addition to multispectral images [80, 81]. This makes Sentinel an excellent alternative for regions where Landsat images find many clouds (such as the Amazon biome), thus filling in any gaps. WorldView images, despite their significant use and very high spatial resolution, are not free, which makes them less accessible.

The best-known satellites for obtaining LiDAR data are ICESat, which collected data from 2003 to 2008 using the Geoscience Laser Altimeter System (GLAS), and the recently launched ICESat-2 containing the Topographic Laser Altimeter System (ATLAS), and the Global Ecosystem Dynamics Investigation (GEDI) [81]. LiDAR technology is one of the most promising for above-ground C quantification: it has the same capacity as forest inventories with high estimation accuracy and low data uncertainty [60, 63]. The main disadvantages of this LiDAR-satellite technology are atmosphere noise, which requires a lot of processing before image using, the discontinuity of the data, and the short operation period [81].

Multispectral, hyperspectral, radar, and laser sensors can also be used in aircraft and drones [53, 63, 66]. Transported lasers are increasingly used due to their remarkable ability to accurately capture canopy height, width, and density [81], allowing reach to remote areas at any time of the year and obtaining a much more accurate special resolution compared to satellites mainly due to the low height of the flights [82]. Despite these advantages, care must be taken with flight height because errors have been found in the equipment’s point cloud for very distant or highly close heights [83].

While LiDAR technology has its advantages, it also has its limitations. Terrestrial LiDAR, for example, can generate an intense point cloud that can be classified into trunks, branches, and leaves [81], and thus it can indirectly estimate the volume and height of trees at ground level [84]. However, it still struggles with very dense vegetation, echoes from the treetops, and noise, making it more suitable for small, more open areas [83]. It’s important to be aware of these limitations when considering its use.

Despite its intense use and technological advances, C quantification by remote sensors is still carried out indirectly, and more information is needed for accurate quantification. LiDAR sensors act as an indirect forest inventory. In contrast, other sensors are commonly used to produce geo-environmental parameters such as meteorological, geomorphological, pedological data [65]; land use and land cover classifications [71 85]; and vegetation indices [54, 66].

[66] used LiDAR data and optical images coupled to the aircraft, together with satellite radar data, both individually and combined. LiDAR alone produced better individual results, while the combination of satellite radar images with LiDAR data produced the best combination, showing its superiority over optical images. Despite achieving good results with optical images, LiDAR data could generate better biomass and C quantification results [86]. Therefore, despite the effectiveness of individual remote sensors, combining different sensors can generate more accurate results with lower uncertainty rates.

Remote sensing is a crucial tool for carbon quantification both above and below ground, mainly because of its ability to cover large areas with a quick revisit time. When integrated with another methodology, such as multispectral, radar, LiDAR and hyperspectral sensors, it can provide increasingly accurate results. However, this data is usually applied to environmental models or regression and machine learning techniques, and therefore requires large amounts of sample data for training, which is one of the biggest obstacles to this type of approach [81]. Platforms that exist in the cloud and have large amounts of images and data available, such as Google Earth Engine, are extremely important and valuable for their ability to facilitate and streamline work while generating global data [87, 88]. With their use, and the promise of new satellites that are more robust and provide more information, we can expect great opportunities for carbon quantification in the coming years.

##### Regression and machine learning

Organic C can be quantified using statistical modeling if the data is parameterized and validated for different land uses, vegetation, and soil types [5]. Regression models, whether with one variable or more, are commonly used to quantify biomass and C because the variables used in the model usually correlate highly with biomass and C that has already been measured [81]. Machine learning techniques are widely used to quantify biomass and C, with the great benefit of using data from remote sensing, even if they are not linearly correlated with biomass and C [81].

Regression models that correlate vegetation and soil data collected and analyzed in the laboratory with data extracted from remote sensing, such as spectral bands, vegetation indices, and topographic variables, can provide a strong correlation among predictor variables and biomass and C [86, 54]. According to [89], machine learning techniques combined with satellite data are also very efficient, as demonstrated in the work of [55, 88], who highlight the use of Random Forest and multispectral, topographic and climatic variables for SOC modeling [90]. used machine-learning techniques to quantify C in soil and vegetation. They highlighted their potential and how the variables used can influence the bias of the models. In addition, regression techniques present accurate results and are an essential methodology for C quantification that will continue to be used due to their ease of reproduction. However, machine learning techniques have frequently appeared in C quantification studies, as shown in Fig. 7. The efficiency of machine learning techniques, especially Random Forests, the most widely-used model for C quantification, is extremely valuable for obtaining more accurate results and helping us estimate the amount of C [90]. The extrapolation capacity of these techniques is also an alternative for quantifying C in different areas, as long as the appropriate parameters and variables are used for each region, which directly influence the capacity and accuracy of models using any machine learning.

Even with all the efficiency and technology of these models, they still have points for improvement, usually relating to the degree of uncertainty generated [3, 81]. state that the main causes of uncertainty in the modeling process are the input data, the model structure, and the model parameters. Input data can have biases and uncertainties from the moment it is collected, whether they are samples collected in the field or satellite image products that suffer from electromagnetic interference and pre-processing. Multiple instances of uncertainty arise when using two or more of these date sources, especially if image scales are not used to reference the actual field scale. Model structures are also subject to uncertainties, such as the choice of model to be used, which may not capture all the complexity of the data or may suffer from overfitting. Model parameters include the number of samples used, multicollinearity between variables, the different parameters used in the models (such as the number of trees in random forests), the weight of connections in neural networks, and more [81]. point out that although it is a major challenge, it is extremely important to quantify these uncertainties and try to negate them by understanding the models used and making use of well-defined and collected variables.

##### Forest inventories

Forest inventories and allometric equations are the best-known and most widely used techniques for quantifying above-ground C. Having been utilized for several decades, they are an essential data source for biomass and C estimation [14]. However, these methods require extensive fieldwork, often involving multiple field trips, which can be labor-intensive and logistically challenging, especially in remote or dense forest areas.

Forest inventories can provide a complete representation of forests, though several and challenges must be considered, including the number of data collected (e.g., number of individuals, collection site, measurements used) and sampling size [15], and variability in tree structure across different species and regions. The high biodiversity in some forests, such as tropical regions, increases this variability, making it more difficult to standardize measurements. The main information used in forest inventories is vegetation type [25, 54], tree age [63, 25], and diameter at breast height (DBH) [65, 15, 71]. Despite the great importance of DBH for calculating biomass, variation in the criteria used to record it can also contribute to inconsistencies in carbon estimates. For example [15], only used trees with DBH greater than 30 cm, while [71] used trees greater than 1.3 m and with DBH greater than 5 cm. Some authors still only use this data to quantify biomass and C [24, 91], thus demonstrating the importance of this data and its correct and mandatory measurement.

Inventory data is used to quantify biomass and C through allometric equations, correlating vegetation data with the amount of C. Different equations have been established for different vegetation types using specific average standards for each vegetation [4]. Forest inventories should be updated to analyze the evolution of vegetation and C capture, using at least measurements of the number of trees and DBH [4]. However, it is critical to ensure that sampling is done at a sufficient scale and frequency to capture the variability in tree species, age, and growth rates. Smaller sample sizes or inadequate site selection can lead to skewed results or misrepresentation of carbon stocks. For example, forest inventories carried out for more than 10 years in China’s subtropical forests have been used to predict the rate of C change over time and confirm the importance of forests for C sequestration and the ability of younger forests to expand sequestration [1]. Such data help to design forest preservation, restoration strategies, and management policies.

Although specific allometric equations are usually used for each vegetation type [86, 15], generalized equations can be used, especially in highly variable environments [81]. For instance, the equation [92] developed can be used for any tropical forest. These generalized equations are essential for analyzing regions with high plant diversity, such as the forests of South America. However, the complexity of forest structure in biodiverse regions can result in less accurate predictions of carbon stocks when generalized equations are applied, as they may not account for the diversity of species and tree forms. Allometric equations can also determine below-ground organic C. For example [64], used an allometric equation to calculate the C ratio between roots and aerial parts of plants.

Forest inventories can be used as primary or secondary data for C quantification [15], or to validate new technologies such as remote sensing [5]. Remote sensing techniques can also estimate vegetation data obtained from forest inventories. However, achieving high accuracy with remote sensing methods remains challenging due to factors such as data noise, the complexity of processing, variations in mapping scale, and operational costs. Additionally, the integration of remote sensing data with ground-based inventories can be difficult in forests with highly variable vegetation structures, further complicating carbon estimation.

### Comparing the methods

The comparison between carbon quantification techniques (Table 1) revealed distinct characteristics between direct and indirect methods, as well as limitations and potentialities that influence their applications.

**Table 1:** Comparison of carbon quantification methods

Technical	strengths		weaknesses	cost	resolution	scalability	technical requirements
WB		Simplicity, low cost, widely used	Incomplete carbon oxidation, toxic reagents, dependence on conversion factors	Low	Low (does not detail all types of carbon)	High (easily applicable in the laboratory)	Requires chemical reagents and basic technical expertise
EA		High precision, fast, complete decomposition of carbon, reference standard	High cost of equipment, specialized maintenance required	High	High (quantifies total carbon accurately)	Medium (requires sophisticated equipment)	Requires specialized equipment and advanced technical knowledge
Gravimetric		Non-destructive technique, avoids the use of chemical reagents	Results influenced by soil mineralogy	Low	Medium (sensitive to mineral variations)	High (widespread in research environments)	Requires strict temperature control and basic equipment for ignition
Spectroscopy		Fast, efficient, field analysis possible, high cost-benefit ratio	Need for multivariate calibration, dependence on spectral libraries	Variable	High (spectra such as MIR offer high precision)	Medium-High (portable devices available)	Requires advanced spectral calibrators and environmental variables.
Remote Sensing		Coverage of large areas, relatively low cost, use of free data	High uncertainty, dependence on additional data for accuracy	Low-Medium	Medium-High (depending on the type of sensor and satellite resolution)	High (applicable in global studies	Requires satellite images and remote analysis systems
Machine learning		High efficiency, extrapolation on different scales, accurate results	Requires large volumes of data for training and validation	Medium	High (adjustable model with different data)	High (versatile for different environments)	Requires large volumes of data and robust computing platforms.
Forest Inventory		Robust method, essential for validating technologies such as LiDAR	Intensive field work, sensitive to structural variations in biodiverse forests	Medium-High	High (given by direct measurement of trees and biomass)	Medium (limitations in remote and biodiverse areas)	Requires detailed physical measurements and field teams.
Geo-environmental models		Simulate environmental carbon processes and evaluate ecosystem services	Can present high uncertainty depending on the availability and quality of data	Variable	High (based on integration of environmental data)	High (applicable to global areas with complex variables)	Requires varied inputs such as meteorological and satellite data

WB and EA are widely used to measure below-ground carbon, standing out for their accessibility and accuracy, respectively, while forest inventories remain essential for quantifying above-ground carbon, despite their labor-intensive nature. Indirect methods, such as spectroscopy, remote sensing, and machine learning, show high potential for scalability and efficiency [93], but face challenges related to dependence on large volumes of data and uncertainty in the models. The availability of global spectral libraries, the advancement of integrative platforms such as Google Earth Engine, and the combination of sensors such as radar and LiDAR represent significant opportunities to improve the accuracy and applicability of these technologies. Each methodology has unique benefits and limitations, highlighting that the integration of direct and indirect approaches, combined with technical improvements, is fundamental to overcoming cost barriers, regional variability, and uncertainties in the results, promoting advances in the estimation of carbon stocks in different ecosystems and global contexts.

### Carbon and land cover classes

Forest was the most studied land cover class, comprising 43.1% of the studies. Forests plays a critical role in CS due to their capacity to store large amounts of C and their biodiversity, which influences this capacity [56, 94]. highlight that carbon dynamics in forests are complex, encompassing not only carbon flow and storage but also economic factors related to deforestation and the broader social and political contexts tied to forests, climate change, and CO_2_ emission reductions that vary across the globe. To combat carbon emissions and climate change resulting from deforestation and environmental degradation, the UNFCCC created the international REDD+ (Reducing Emissions from Deforestation and Forest Degradation) initiative, which is aimed not only at combating carbon emissions, but also at increasing conservation, sustainable management, and carbon stocks in forest areas. The importance of protecting these forests is that, according to [95], deforestation contributes 12–17% of global greenhouse gas emissions.

Forests are also the most frequently converted land cover, often replaced by pasture, agricultural lands, or deforested ecosystems [4]. Such land cover changes are among the primary causes of CO_2_ emissions [94, 85]. Agriculture, the second most studied land cover class, similarly impacts carbon dynamics. The conversion of forests to agricultural areas influences soil organic carbon (SOC), which can either absorb or release C depending on crop type and management practices [85]. Although the extent of carbon sequestration in agricultural systems varies based on climatic, biophysical, and management factors [94], studies overwhelmingly show that forest ecosystems sequester more carbon compared to agricultural lands. Effective land management, particularly in pastures, can contribute to carbon capture, but these systems currently do not match the carbon sequestration potential of forests [85].

Less prominent landscapes like mangroves, peatlands, and wetlands are increasingly being recognized for their critical role in carbon sequestration [96, 46, 97]. These ecosystems are considered carbon hotspots by the IPCC due to their ability to store significant amounts of carbon. Peatlands, for instance, store large quantities of carbon, and their drainage for agricultural or pastoral use can release this stored carbon into the atmosphere [4, 47]. state that peatlands hold between 5 and 20% of all carbon stocks, as well as other ecosystem services, even though they occupy only 3% of the global territory. A major problem with peatlands is their identification, since there are no high-resolution maps that clearly define the location of these environments, which makes it difficult to study and preserve them [47]. Mangroves, particularly those in tropical regions, are also critical ecosystems because they have the capacity to sequester carbon 40x faster than other terrestrial ecosystems [98]. In Bangladesh, mangroves are characterized as the second largest accumulator of carbon in natural areas [72].

Although most studies focus on forests, because they are the most visible and more common, we need to understand that every system has the capacity to store carbon. Transitions in land use, one of the greatest challenges in climate change mitigation, not only impact forests but also threaten the integrity of these lesser-known carbon hotspots. The delineation, preservation and restoration of these regions must be a priority in all carbon and global change mitigation scenarios.

## Conclusion

Carbon management, especially its storage and absorption in the soil, is of vital importance to society, given its impact on agricultural sustainability and climate change control. Although scientific publications have increased, especially since 2013, this increase is concentrated in developed countries, highlighting the lack of investment in research and technology in countries located in the Southern Hemisphere.

Carbon quantification techniques have evolved significantly, moving from traditional methods such as Walkley-Black (WB) to modern alternatives such as Elemental Analysis (EA), which have less environmental impact. In addition, technologies such as remote sensing, spectroscopy, and environmental modeling based on machine learning have gained prominence due to their low cost, wide availability of free data and ability to extrapolate. Despite this, older techniques such as forest inventories are still widely used, either because of their reliability or because of the need to validate more recent models, including LiDAR and machine learning.

Given the advancement of these new technologies, there are important challenges and opportunities for the future of carbon quantification research. This includes the integration of data from multiple sources (e.g. UAVs, satellites and in situ spectroscopy), allowing the combination of different approaches to achieve more accurate estimates. In addition, the development of global spectral libraries for SOC estimation can improve the replicability and harmonization of methodologies between different regions. Investments should be directed towards increasing the transferability of machine learning models between regions and ecosystems, optimizing their global application. It is also essential to address the uncertainties and reproducibility of the models, ensuring that the results obtained are more reliable and have practical applicability.

Finally, understanding carbon fluxes in plants and soils, along with strengthening policies and practices to mitigate climate change, will continue to depend on increasingly refined and integrated technologies. Investments in long-term research and robust data can accelerate this evolution, enabling substantial advances in sustainable carbon management and tackling global climate change.

## Data Availability

No datasets were generated or analysed during the current study.

## Abbreviations

C: Carbon
CS: Carbon Stock
EA: Elemental Analysis
LULC: Land Use Land Cover
MIR: Mid Infrared Spectrum
NIR: Near Infrared
PRISMA: Preferred Reporting Items for Systematic Review and Meta Analyses
SC: Soil Carbon
SOC: Soil Organic Carbon
SWIR: Shortwave Infrared
USA: United States of America
VIS: Visible Spectrum
WB: Walkley Black
